# Antimicrobial activity and pH measurement of calcium silicate cements versus new bioactive resin composite restorative material

**DOI:** 10.1186/s12903-019-0933-z

**Published:** 2019-11-04

**Authors:** Ashraf Abou ElReash, Hamdi Hamama, Waleed Eldars, Gong Lingwei, Ahmed M. Zaen El-Din, Xie Xiaoli

**Affiliations:** 1 Department of Endodontic, Xiangya School of stomatology, Central South University, Xiangya Road No 72. Kaifu, Changsha, 410078 Hunan province China; 2 Department of Operative Dentistry, Faculty of Dentistry, Mansoura University, Mansoura, Egypt; 3 Department of Medical Microbiology and Immunology, Faculty of Medicine, Mansoura University, Mansoura, Egypt; 4 Department of Restorative Dentistry, Faculty of Oral and Dental Medicine, Delta University for Science and Technology, International Coastal Road, Gamasa, Mansoura, Egypt

**Keywords:** Antimicrobial, Bioactive composite, Calcium silicate, pH, MTA

## Abstract

**Background:**

The purpose of this in vitro study is to compare the antimicrobial effect and pH of two calcium silicate cements Mineral trioxide aggregate high plasticity (Angelus PR, Brazil) and iRoot BP Plus (BioCeramix Inc., Vancouver, BC, Canada) and new bioactive restorative resin composite restorative material (ACTIVA, MA, Pulpdent, USA) against aerobic bacteria, strictly anaerobic bacteria and a yeast by using an agar diffusion test.

**Methods:**

The materials were tested immediately after manipulation and were applied to the agar plates. Sodium hypochlorite (NaOCl) 5.25% was used as a positive control group. The dry filter paper acted as a negative control group for this study. The size of the inhibition zone for each material was measured after 12, 24 and 48 h. At the time of pH measurement; materials were prepared, crushed then dispersed in distilled water.

**Results:**

The one-way Anova test revealed that iRoot BP Plus significantly showed superior antimicrobial efficacy compared to MTA-HP against the following species; *Staphylococcus aureus, Streptococcus mutans, Enterococcus faecalis, Enterococcus faecium, Peptostreptococcus anaerobius and Candida albicans (P < 0.05).* All of the tested materials did not show any antimicrobial effect against *Porphyromonas gingivalis* and *Actinomyces israelii.* The new bioactive resin composite material (ACTIVA) showed the least antimicrobial activity against the previously mentioned microorganisms except *E. faecalis.* NaOCl significantly showed the highest antimicrobial activity among the test group (*P* < 0.05). iRoot BP Plus was more alkaline (pH 12.1 ± 0.14/ 11.9 ± 0.25) in comparison to MTA-HP (pH 11.6 ± 0.16/ 11.2 ± 0.10) while ACTIVA was slightly acidic (pH 5.4 ± 0.09/ 6.5 ± 0.08).

**Conclusions:**

According to the findings of this study, it was concluded that calcium silicate- based cements showed a potential antimicrobial activity mainly due to its high alkalinity. The new bioactive resin composite restorative material exhibits less antimicrobial activity due to its resinous ingredients and slightly acidic nature. Antimicrobial effect of calcium silicate cements against strictly anaerobic bacterial species is still questionable.

## Background

Bacteria and other microorganisms are considered to be the main causative factors of all endodontic diseases [1, 2]. Even with good root canal treatment, remaining microorganisms can survive in the lateral dentinal tubules and apical ramifications [3, 4], thus endodontic failure does not take place unless microorganisms invade the damaged area; and establish a progressive tissue breakdown [5].

The oral cavity contains more than 500 microbial species capable of invading the root canal system. Among the identified species, there are aerobic- facultative bacteria; *(*e.g. *E. faecalis*, *E. faecum, S. mutans* and *S. aureus)* and strictly anaerobic bacteria (e.g. *P. gingivalis*, *A. israelii* and *P. anaerobius)*, and *C. albicans* yeast*.* The previously mentioned specimens are considered the most resistant in oral cavity and several studies [6–13] reported them as one of the most important etiological factors for root canal treatment failures.

One of the most imperative properties that restorative material should have beside its biocompatibility and sealing ability, is the antimicrobial activity and prevention of ingress and survival of microorganisms. This can influence the success of the treatment [14]. Several restorative materials are available in the market such as amalgam, composite resin, zinc-oxide improved cements (IRM; Dentsply, USA; Super-EBA; Bosworth Co, USA) cements, glass ionomer cements and MTA [15]. Despite the great success achieved by MTA, the disadvantages related to long setting time, discoloration and poor handling properties resulted in the need of necessary improvements [16, 17].

A new formula of MTA was developed to overcome the previously mentioned disadvantages. It was introduced into the markets under the name “MTA - HP” (Angelus PR, Brazil). This new calcium silicate-based cement has calcium tungstate as a radiopacifier, shorter setting time, low solubility and superior handling properties. Moreover, no discoloration was observed after the removal of bismuth oxide. This improvement in properties is attributed to the new powder formula in addition to an organic plasticizer to the liquid [18, 19]. Another trial was made to improve the older “powder – liquid” form of MTA. It was introduced in a premixed readymade paste, “iRoot BP Plus” Root Repair Material by Innovative BioCeramix Inc., Vancouver, BC, Canada, which is a totally synthetic calcium silicate-based cement with bioceramic nanotechnology. It was thought to be a better alternative to traditional MTA in terms of biocompatibility, antimicrobial activity and higher physical properties [20].

A new bioactive resin composite restorative material “ACTIVA” was developed as a combination between the aesthetic and high physical properties of composites; moreover, it can release and recharge calcium, fluoride and phosphate ions of glass ionomer and resin modified glass ionomer (RMGI), therefore it is considered an enhanced RMGI [18]. The manufacturer claims that ACTIVA Bioactive restorative resin composite (ACTIVA, MA, Pulpdent, USA) has exceptional ionic shock absorbing resin matrix, infiltrated with glass ionomer filler and considered to be the first bioactive resin composite with superior antimicrobial properties. This is due to the dynamic exchange of calcium and fluoride [21].

After extensive research regarding the antimicrobial activity of these new materials, the literature results found were variable. MTA products were extensively investigated in many articles with different methodologies [11, 16, 22]. Few studies [20, 23, 24] were conducted on iRoot products, while none were found for ACTIVA. There was scarcity of information about the comparative evaluation of the antimicrobial activity against certain species to give a full picture for clinical application. Additionally, there is no article comparing their properties, especially in terms of antimicrobial property.

The Agar diffusion test (ADT) was used in this experiment to test the direct exposure of freshly mixed materials. Despite the high accuracy of other testing methods, ADT showed many advantages; simplicity, high reliability, less technique sensitivity and popularity for testing the antimicrobial properties for different restorative materials [25, 26]. Therefore, the aim of this in vitro study was to compare the pH and antimicrobial properties of MTA-HP, iRoot BP Plus and ACTIVA bioactive restorative restorative materials at different time intervals.

This study was designed to test the null- hypothesis to prove there was no significant difference in antimicrobial activity of tested materials in comparison with 5.25% NaOCl solution.

## Methods

All the test materials used for this study are listed in **(**Table 1**)**.

**Table 1:** Materials list

Materials	Constituents	Manufacturer
Mineral trioxide aggregate High Plasticity (MTA-HP)	Powder: BI_2_O_3_, CaO, MgO, K_2_O, Na_2_O, Fe_2_O3, SO_3_, SiO_2_, Al_2_O_3_ Liquid: distilled water with organic plasticizer	Angelus Co.Londrina, PR, Brazil
iRoot BP Plus	Paste containing: Calcium silicates, zirconium oxide, tantalum pentoxide, calcium phosphate monobasic	Innovative BioCeramix Inc. Canada
ACTIVA Bioactive Restorative	Light curing (resin based) Blend of diurethane and other methacrylates with modified polyacrylic acid (∼53.2%), silica (∼3.0%), sodium fluoride (∼0.9%), and so on	Pulpdent, Watertown, MA, USA

### Antimicrobial test: (agar diffusion test)

All the experimental procedures were done under aseptic conditions in laminar air flow (Unilab biological safety cabinet class II, China).

The antimicrobial activity was evaluated using five standard bacterial strains and a yeast: *E. faecalis* (ATCC® 19433™^*^), *E. faecum* (ATCC® 51559™^*^), *S. aureus* (ATCC® 29213™^*^), *S. mutans* (ATCC® 25175™^*^), *P. gingivalis* (ATCC® 3327™^*^) and *C. albicans* (ATCC® 10231™^*^) obtained from Liofilchem® Via Scozia, Zona Industeriale. Italy. Two bacterial strains: *A. israelii* and *P. anaerobius* were isolated from the infected root canals from outpatients recruited from Endodontic Department clinics, Xiangya Stomatological Hospital, Central South University, China during routine endodontic treatment. The collected microorganisms were identified by using conventional biochemical tests*.* All the previously mentioned clinical/ laboratory steps were approved by the ethical committee of Central South University (No: 20180025). Bacterial suspension was prepared for each bacterial strain and the turbidity was adjusted against 0.5 ml McFarland solution. *S. mutans, E. faecum, P. anaerobius, A. israelii, P. gingivalis and C. albicans* were streaked on 10% sheep Blood- Agar plates (Oxoid. U.K) plates, while *E. faecalis and S. aureus* were streaked on Mueller-Hinton Agar plates (Oxoid. U.K). A lawn of growth was achieved using sterile cotton-tipped swab inoculating the bacterial suspension onto the agar plate. Thereafter, 5 equidistant wells with a diameter of 5 mm and a depth of 5 mm were made in each plate (total 40wells in 8 plates for each bacterial strain) by removing the agar with a sterile hand tissue punch (TPH50S. OSUNG.USA).

The tested materials were manipulated with sterile instruments according to the manufacturer’s instruction. Freshly prepared materials were placed into the wells of customized sterile teflon template 5 mm thickness with standardized holes 5 mm diameter, after setting material disks were removed from the template and pressed into place in the agar plate using a sterile carrier.

NaOCl 5.25% was used as a positive control, whereas sterile dry filter papers not impregnated with any material were used as the negative control. The three strict anaerobic microorganisms were incubated in anaerobic gaseous conditions inside McIntosh filde’s anaerobic culture jar, with gas packs of Anaerogen® 2.5 L (Oxoid. U. K). All plates were maintained at room temperature for 2 h, to allow diffusion of the test materials and then incubated at 37 °C for 48 h in an incubator.

The diameter of the halo formed around the materials (inhibition zone) was measured by the same operator in two perpendicular locations with a millimetre ruler (sliding calliper) with an accuracy of 0.5 mm, after 12 h, 24 h and 48 h. The size of the inhibition zone was calculated as follows:

Size of inhibition zone = (diameter of halo − diameter of specimen) × ½.

All the assays were conducted in triplicate and the results were recorded in terms of the average diameter of the inhibition zone [27, 28].

### Measurements of pH

Each material is prepared and left until full setting, then crushed and mixed with distilled water to form a Suspension at a concentration of 50 mg/ ml. Then the samples were centrifuged for 30 s to provide a clear supernatant. A pH meter (ADWA AD1030®- Hungary) was used to measure the pH of the supernatant of each material. The results were calculated immediately and after 60 min. Distilled water was used as a control. The mean values of pH with the standard deviation were calculated.

### Statistical analysis

The data was analysed with one-way analysis of variance and the Tukey’s post hoc test for multiple comparisons between the antimicrobial effects of the three root repair materials against each bacterial strain tested. The level of significance was established at 5%. Statistical analysis was performed with SPSS software (IBM, Armonk, NY, USA).

## Results

### Antimicrobial activity

**Fig. 1:**
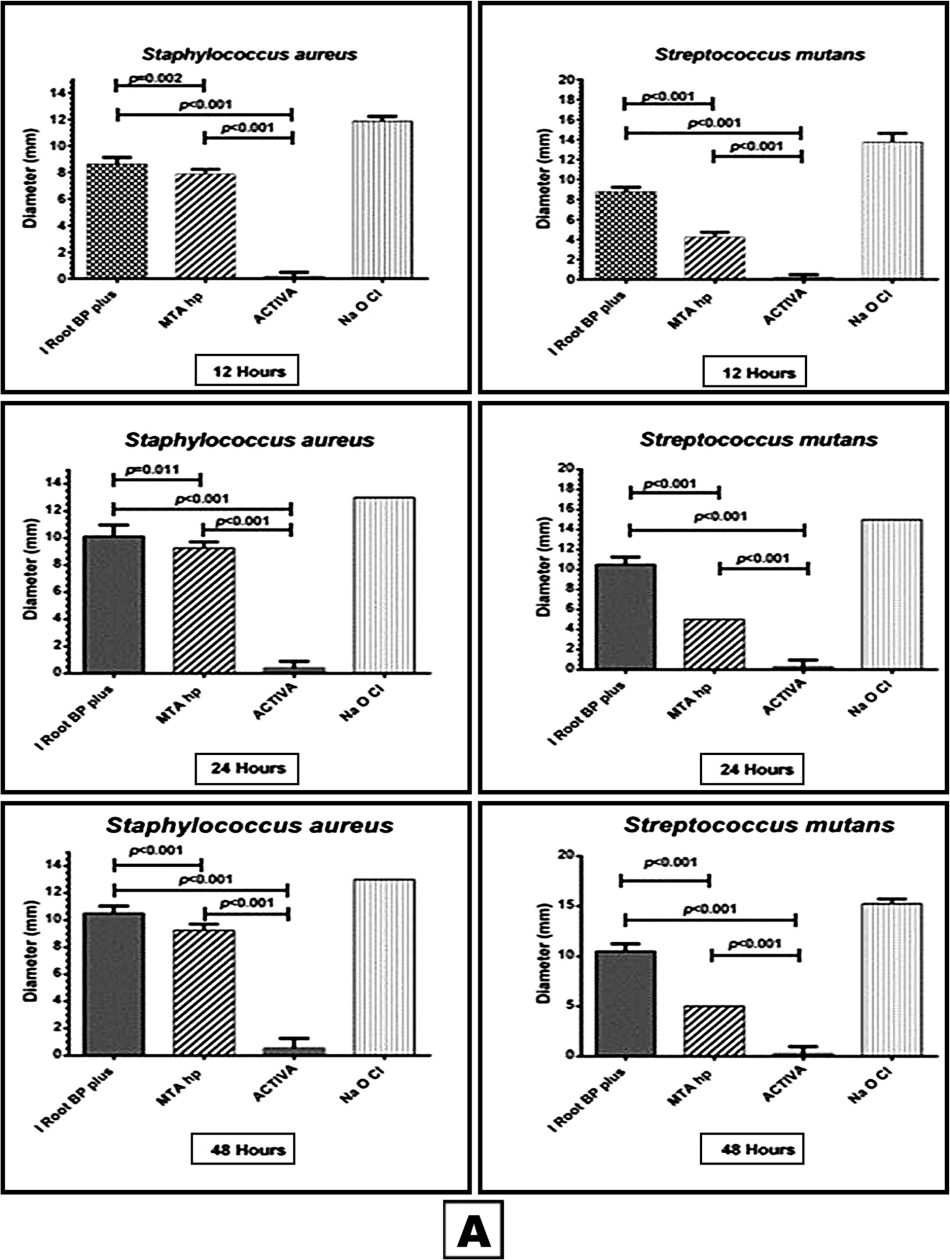
This box and whisker with stem is showing the antibacterial effect of the three materials (iRoot BP, MTA- HP and ACTIVA) and NaOCl (positive control) against *Staphylococcus aureus* and *Streptococcus mutans*

**Fig. 2:**
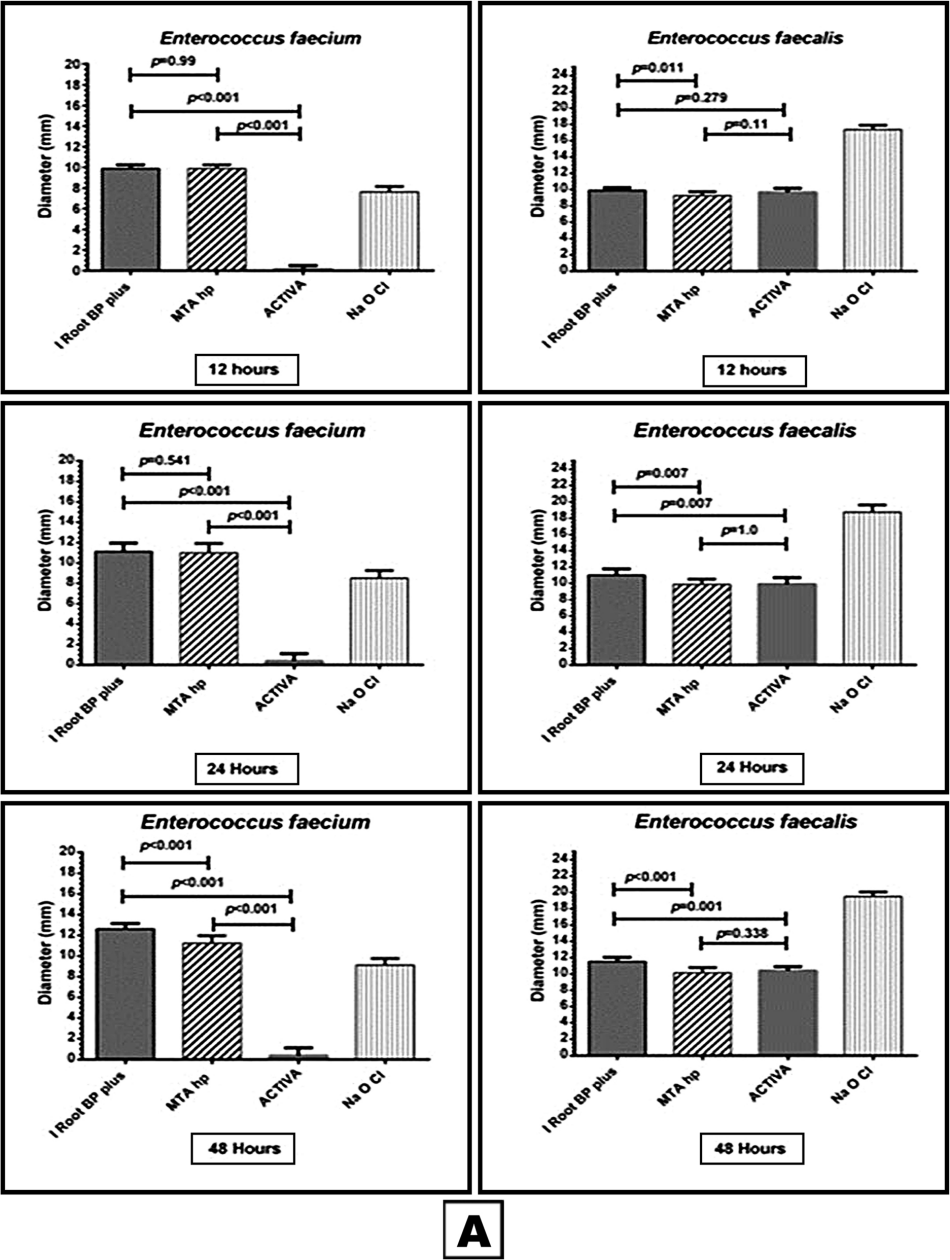
This box and whisker with stem is showing the antibacterial effect of the three materials (iRoot BP, MTA- HP and ACTIVA) and NaOCl (positive control) against *Enterococcus faecium* and *Enterococcus faecalis*

Concerning Gram +ve facultative aerobic cocci (*S. aureus*, *S. mutans, E. faecalis and E. faecium)* iRoot BP and MTA- HP had significantly inhibitory effect on *S. aureus*, *S. mutans* and *E. faecium* (Figs. 1 and 2) in all time periods compared to ACTIVA (*P < 0.001)*. The inhibitory effect of i Root BP was significantly higher than MTA- HP when compared to each other *(P < 0.05).* On other hand all the three materials had antimicrobial effect on *E. faecalis* but the best effect was exerted by i Root BP (Fig. 2).

**Fig. 3:**
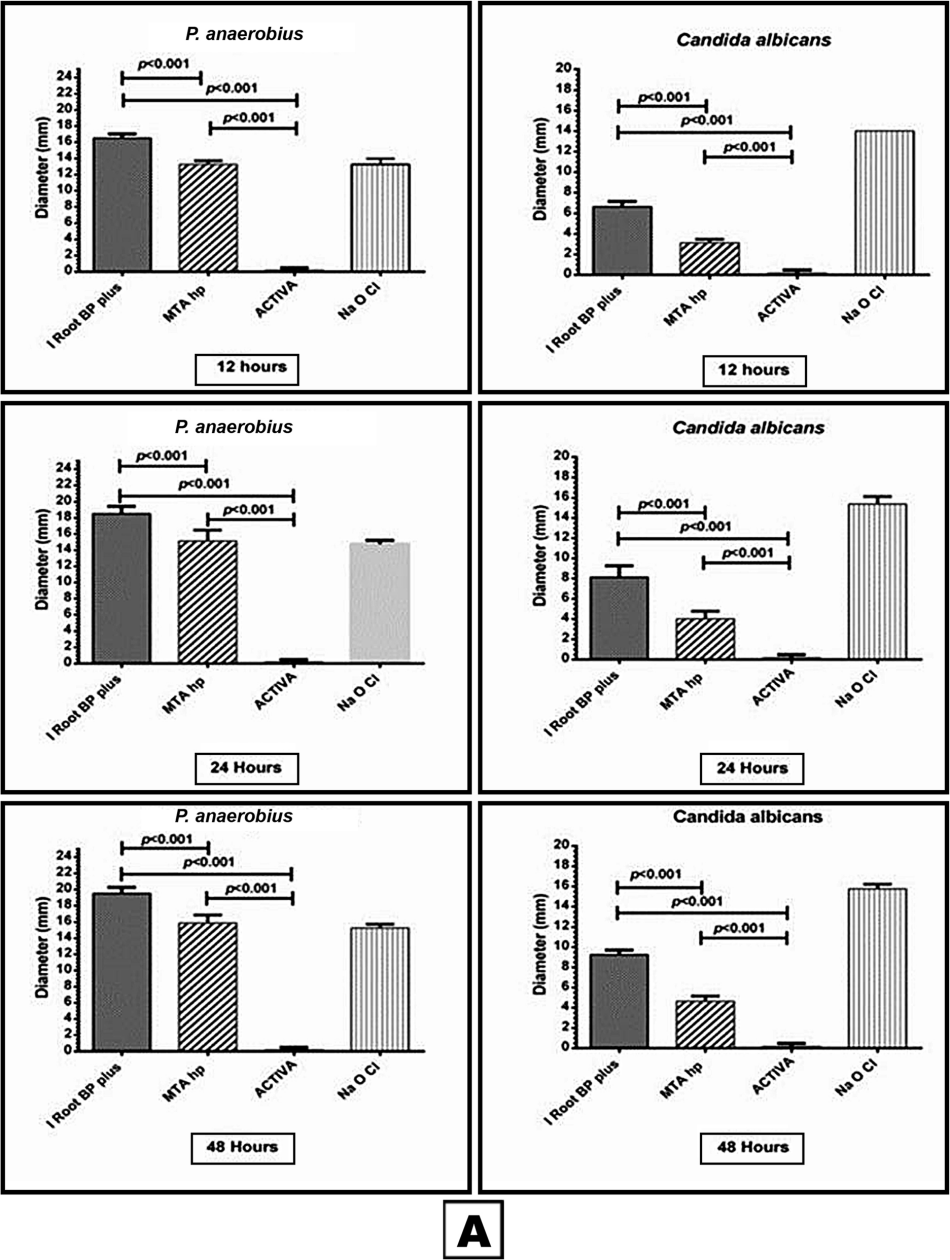
This box and whisker with stem is showing the antimicrobial effect of the three materials (iRoot BP, MTA- HP and ACTIVA) and NaOCl (positive control) against *Peptostreptococcus anaerobius* and *Candida albicans*

Meanwhile the results of anaerobic Gram +ve and Gram -ve bacteria *P. anaerobius, A. israelii and P. gingivalis* showed again i Root BP and MTA- HP had inhibitory effect on *P. anaerobius* in comparison to ACTIVA which had no effect at all (*P < 0.001)* with also significantly better effect of i Root BP when compared to MTA- HP (*P < 0.001)* (Fig. 3). Unexpectedly none of the three materials had any antimicrobial effect on both *A. israelii and P. gingivalis.*

Finally, MTA- HP had better inhibitory effect on *C. albicans* than i Root BP (*P< 0.001)* while ACTIVA didn’t have any antimicrobial effect at all time periods (Fig. 3).

### pH measurements

**Fig. 4:**
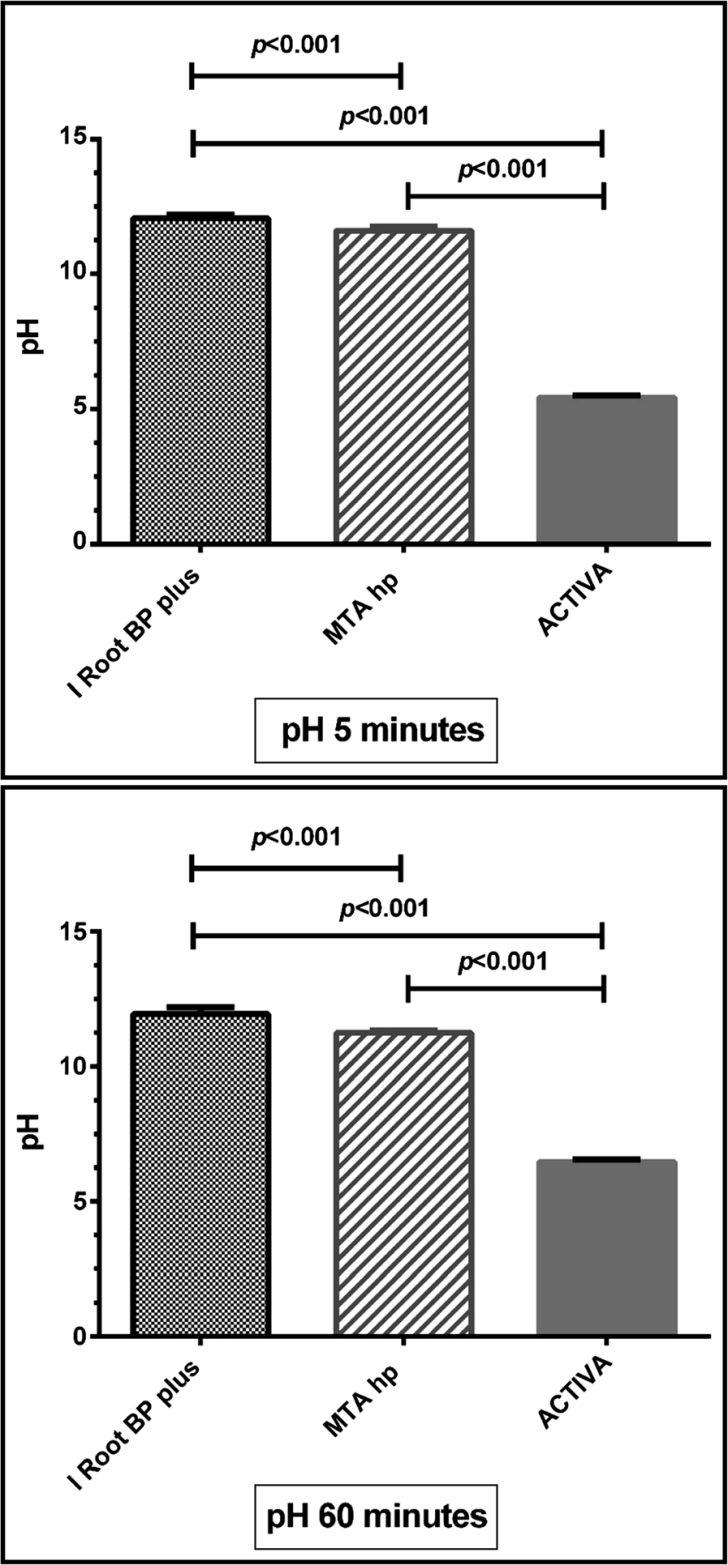
This box and whisker with stem is showing the pH measurement changes of the three materials (iRoot BP plus, MTA- HP and ACTIVA) after 5 min and 60 min respectively

As shown in (Table 2) and (Fig. 4) ACTIVA exhibited very weak acidic effect with lower pH level (5.4 ± 0.09 and 6.5 ± 0.08) after 5 and 60 min respectively. In contrast iRoot BP showed significantly strong alkaline effect with higher initial pH measurements (12.1 ± 0.14 and 11.9 ± 0.25) when compared to MTA- HP (11.6 ± 0.16 and 11.2 ± 0.10) after 5 and 60 min respectively.

**Table 2:** Mean and standard deviation of pH measurements after 5 and 60 min

	MTA HP	iRoot BP plus	ACTIVA
pH 5 min (mean ± SD)	11.6 ± 0.16	12.1 ± 0.14	5.4 ± 0.09
pH 60 min (mean ± SD)	11.2 ± 0.10	11.9 ± 0.25	6.5 ± 0.08

## Discussion

The ultimate goal of endodontic treatment outcome is the reduction or elimination of poly-microbial environment in pulpal and peri-radicular infections [29]. The present study tested the antimicrobial properties and pH values of different endodontic repair materials. The agar diffusion test was used in this study for evaluation of the antimicrobial activity against a collection of facultative, aerobic, strictly anaerobic bacteria and a yeast which are popular species of perio-endodontic pathogens [30].

The selection of the included materials “iRoot BP, MTA- HP and ACTIVA” was based on the scarcity of literature regarding their antimicrobial properties, and were compared to MTA products which were extensively researched and had reliable results [11, 16, 22]. The agar diffusion test is considered to be the standard assay for initial screening of antimicrobial activity. It is the most convenient method due to its simplicity to be performed and credibility to be used in testing the antimicrobial properties of freshly mixed materials. Although the direct contact test method is more recent, it is more challenging to be performed. It requires meticulous steps to be carried out in sensitive environment conditions; moreover, it is more expensive in comparison to ADT. It was difficult to be performed under the current study conditions [31].

The selection of the used bacterial species in this study was intended to represent the poly-micro flora in the oral cavity. Facultative bacteria, such as *E. faecalis*, *E. faecum*, aerobic bacteria like *S. aureus, S. mutans*, and strictly anaerobic bacteria, including *P. gingivalis*, *A. israelii* and *P. anaerobius*, in addition to a yeast “*C. albicans* “.

Our results showed that the inhibition zones observed for MTA- HP and iRoot BP were comparable to each other with superior performance of iRoot BP. They had antimicrobial activity against all tested microorganisms except *A. israelii* and *P. gingivalis*. Unexpectedly both calcium silicate-based cements had antimicrobial effect against *P. anaerobius*. While ACTIVA didn’t show any antimicrobial activity except against *E. faecalis*.

Calcium hydroxide is a major output of MTA and iRoot products. When it reacts with water or body fluids resulting in an increase of the pH of the media turning it into an alkaline medium [32]. According to the results of our experiment MTA- HP and iRoot BP had high initial alkaline pH, which was in accordance with the findings of Torabinejad et al [16] regarding MTA, so it was assumed that iRoot would have the same characteristics as it’s calcium silicate-based cement. Hereby, high alkaline pH and hydroxyl ions were found to affect the cell membrane and enzymatic activity of microorganisms as discovered by Estrela et al [33].

The combination of calcium hydroxide diffusion with high alkaline pH of MTA- HP and iRoot BP can explain their antifungal and antimicrobial effect against *C. albicans*, *S. aureus, S. mutans,* and *E. faecalis*, which are in consistence with the findings of previous studies [11, 23, 24, 34, 35]. The higher initial pH values of iRoot BP than MTA- HP can explain its superiority in the antimicrobial activity. Moreover, Damlar et al [20] found that MTA and iRoot BP Plus exhibited similar antimicrobial properties against *E. faecalis* and *C. albicans*. In addition McHugh [36] stated that high alkaline pH more than 11.5, is bactericidal to *E. faecalis*.

The prevalence and ability of survival of *P. gingivalis* and *A. israelii* in oral infections are much higher than *Peptostreptococcus anaerobius* [37]. Negative results against *P. gingivalis* and *A. israelii* are supported by the results of Odabaş et al [38] who found that MTA alone does not have antimicrobial activity against *A. israelii* but, after addition of silver-zeolite showed enhanced antimicrobial effect due to the antimicrobial property of silver; On the other hand Ryan et al [28] recorded some inhibitory effect of MTA angelus on *P. gingivalis*. This can be explained by the reactive oxygen layer created on the surface of MTA when exposed to aerobic conditions during preparation, which will have antimicrobial effect, while the anaerobic conditions of our study will prevent the elaboration of these free radicals. This will not inhibit growth of anaerobic species [22, 39].

Regarding the effect of MTA- HP and iRoot BP on *P. anaerobius*, it was explained by Byström et al [40] that *P. anaerobius* was killed after 1 min exposure to saturated calcium hydroxide solution. This was in contrast with the findings of Torabinejad et al [11] who reported that MTA had no antimicrobial activity against anaerobic bacteria. MTA has been tested in many researches but with contradictory results [16, 41–44]. This may be attributed to the usage of different methodologies, bacterial strains, aerobic and anaerobic conditions.

The result of this experiment showed that ACTIVA had very weak antimicrobial activity against all strains tested except *E. faecalis*. The reason may be due to the antimicrobial potentiality of ions released from resin based materials directly proportioned to the drop in pH levels [45].

In light of this study outcome, the weak acidity of ACTIVA is attributed to the presence of modified polyacrylic acid within the component of this bioactive resin composite based material (Table 1). Moreover the resinous components of ACTIVA (Diurethane and other methacrylates) which adversely affect the diffusibility of the material through the agar, and the material interaction with the microenvironment as described by Matalon et al. [46]. They found that the polymerization of composite can affect its antimicrobial activity, although the antimicrobial effect of its original components; In addition, a pH level lower than 4.8 was optimum to inhibit *S. mutans* growth which cannot be affected by the weak acidic action of ACTIVA of pH 5.5 [47].

On the other hand, the positive inhibition zone of *E. faecalis*, was in accordance with the findings of a previous investigation which assumed that other factors such as the leached out components like unreacted monomers, or photo-initiator products, are responsible for this effect [23]. Gerami-Nejad and Stretton [48] reported a higher antimicrobial effect of diphenyliodoniumchloride (DPICl) on *E. faecalis* more than that observed for *S. mutans*; In addition, fluoride ions may play a role in which fluoride(F^−^) can interact with phosphate groups (PO_4_^− 3^) of lipoteichoic acid (LTA) by electrostatic interaction causing membrane instability of *E. faecalis* [49].

## Conclusion

According to the findings of this study, it was concluded that calcium silicate- based cements showed a potential antimicrobial activity mainly due to its high alkalinity. The new bioactive resin composite restorative material exhibits less antimicrobial activity due to its resinous ingredients and slightly acidic nature. Antimicrobial effect of calcium silicate cements against strictly anaerobic bacterial species is still questionable. Further studies on the development of resistance ability of these microorganisms are needed.

## Data Availability

All the datasets used and analysed during the current study are available from the corresponding author on reasonable request.

## Abbreviations

*A. israelii*: *Actinomyces israelii*
ADT: Agar diffusion test
*C. albicans*: *Candida albicans*
*E. faecalis*: *Enterococcus faecalis*
*E. faecum*: *Enterococcus faecum*
MTA- HP: Mineral trioxide aggregate high plasticity
NaOCl: Sodium hypochlorite
*P. anaerobius*: *Peptostreptococcus anaerobius*
*P. gingivalis*: *Porphyromonas gingivalis*
*S. aureus*: *Staphylococcus aureus*
*S. mutans*: *Streptococcus mutans*
